# Disruption of mitochondrial DNA integrity in cardiomyocyte injury upon ischemia/reperfusion

**DOI:** 10.1016/j.gendis.2024.101282

**Published:** 2024-03-28

**Authors:** Shengnan Hu, Xueying Tang, Fangrui Zhu, Chen Liang, Sa Wang, Hongjie Wang, Peifeng Li, Yuzhen Li

**Affiliations:** a School of Basic Medical Sciences, Hebei University, Baoding, Hebei 071002, China; b Basic Medical Department, Graduate School, Chinese PLA General Hospital, Beijing 100853, China; c Institute for Translational Medicine, The Affiliated Hospital of Qingdao University, College of Medicine, Qingdao University, Qingdao, Shandong 266000, China; d The First Affiliated Hospital of Jinzhou Medical University, Jinzhou, Liaoning 121000, China

**Keywords:** Cardiomyocyte, Ischemia/reperfusion, mtDNA, Package, Repair, Replication, Transcription

## Abstract

Mitochondria serve as the energy provider and enable life activities, and they are the only organelles containing extra-chromosomal DNA. Each mitochondrion contains multiple copies of its genome, which is usually referred to as mitochondrial DNA (mtDNA). mtDNA encodes necessary electron transport chain complex subunits, as well as the essential RNAs for their translation within the organelle. Therefore, the precondition for intact mitochondrial function and cardiomyocyte survival is the integrity of mtDNA. Accumulating evidence suggests that the disruption of mtDNA integrity is involved in ischemia/reperfusion-induced mitochondrial dysfunction and cardiomyocyte injury. Here, we review the current opinions about the pathways of mtDNA integrity maintenance and discuss the role of mtDNA integrity in cardiomyocyte injury reacting to ischemia/reperfusion. We also discuss the mechanisms by which mtDNA mediates ischemia/reperfusion-induced cardiomyocyte injury, together with therapeutic strategies by targeting mtDNA.

## Introduction

Acute myocardial infarction induced by ischemia severely threatens human health and life. Timely reperfusion therapy after ischemia is an effective measure to reduce acute myocardial ischemic injury and inhibit the expansion of infarction. However, as the blood supply of the myocardium recovers, myocardial reperfusion injury known as ischemia/reperfusion (I/R) injury often occurs. It can cause irreversible detrimental effects. A high density of mitochondria is located in cardiomyocytes and are central targets of I/R injury.^1^^,^^2^ Large amounts of functional mitochondria are needed by cardiomyocytes as cardiomyocytes consume plenty of energy. Myocardial mitochondria produce a total of 90% of ATP for normal cardiac functions. Mitochondria are the only kind of organelles that consist of DNA other than chromosomal DNA within the cell (except in plant chloroplasts).^3^ Each mitochondrion contains several sets of its genome, mitochondrial DNA (mtDNA).^4^ mtDNA encodes the necessary subunits of electron transport chain complexes and the essential RNAs for their translation within the organelle.^3^^,^^4^ Thus, mitochondria are under the control of both mtDNA and nuclear DNA. mtDNA is very susceptible to various stimuli.^4^ Previous studies demonstrate that I/R reduces mtDNA replication and transcript levels, and destroys mtDNA package and repair mechanisms, thus disrupting the integrity of mtDNA.^5^, ^6^, ^7^ Once mtDNA integrity is broken, it inevitably results in mitochondrial dysfunction, thereby causing cardiomyocyte injury.

## mtDNA structure and function

Mitochondria are composed of the outer membrane, inner membrane, intermembrane space between the double membranes, and matrix inside the inner membrane. mtDNA is harbored in matrix.^8^ The first complete human mtDNA sequence was identified in 1981.^9^ mtDNA of the yeast *Saccharomyces cerevisiae* was sequenced in 1998.^10^ Interestingly, it was found that the structure and coding function of mammalian mtDNA is different from that of yeast mtDNA (Table 1). Yeast mtDNA is an 8500-base-pair double-stranded linear DNA that contains introns. In contrast, mammalian mtDNA contains 16,569 base pairs and is displayed as a closed circular molecule.^11^ It has two strands that are different in GC content. One is a heavy-strand enriched G and the other is a light-strand enriched C. Mammalian mtDNA contains 37 genes. All genes in mammalian mtDNA have no 5′ or 3′ non-coding sequences and no introns.^12^ Only a 1.1-kb region of mammalian mtDNA is noncoding, also termed as the control region.^11^^,^^12^ The control region consists of a special three-stranded DNA loop structure.^11^^,^^13^ The light-strand promoter together with two heavy-strand promoters is contained in the control region.^12^ Also, in the control region, there are regulatory sequences controlling mtDNA replication. Therefore, the control region is a very important structure of mammalian mtDNA.

**Table 1:** The comparison of human mtDNA with yeast mtDNA.

mtDNA	Human	Yeast
Base pairs (bp)	16,569	8500
DNA conformation	circular DNA	linear DNA
Introns	no	yes
The number of genes	37	34
mtDNA products		
Genes for protein synthesis	COX1 COX2 COX3 ATPase 6 ATPase 8 Cytochrome b ND1 ND 2 ND 3 ND 4 ND 5 ND 6 ND 4L	COX1 COX2 COX3 ATPase 6 ATPase 8 ATPase 9 Cytochrome b Ribosomal protein
Genes for rRNAs	12S rRNA 16S rRNA	15S rRNA 21S rRNA
Genes for tRNAs	22 tRNA	24 tRNA

COX: cytochrome *c* oxidase; ATPase: ATP synthase; ND: NADH dehydrogenase.

**Figure 1:**
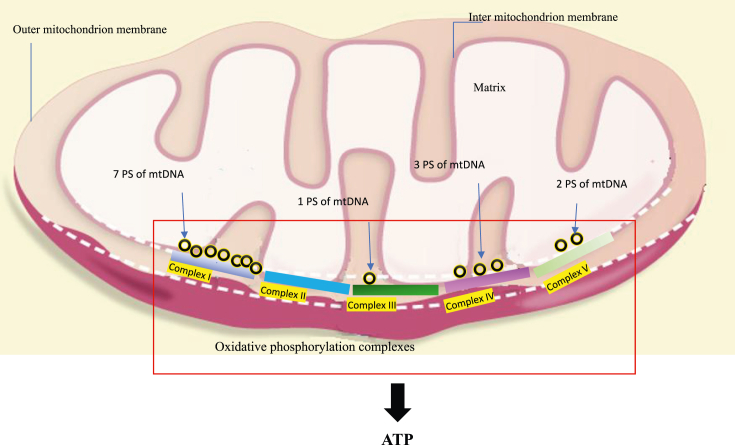
Summary of the effect of mammalian mtDNA on contributing 13 polypeptide subunits (PS) to five oxidative phosphorylation complexes (I–V) that make up the OXPHOS. The inner mitochondrial membrane containing five complexes is where ATP production takes place.

Mitoproteomics studies are performed and reveal that human mitochondria contain about 1500 proteins. Thirteen of these proteins are encoded by mtDNA. Although most of the mitochondrial proteins are not encoded by mtDNA, these mitochondria-encoded proteins play a critical role in maintaining ATP production at normal levels. Thirteen mitochondria-encoded proteins include cytochrome c oxidase subunit I/II/III, two subunits of ATP synthase (ATPase 6, 8), cytochrome b, and NADH dehydrogenase subunit 1–6/4L. Five multi-enzyme complexes I, II, III, IV, and V form the mammalian oxidative phosphorylation system (OXPHOS). All of these mitochondria-encoded proteins function as structural subunits of multipolypeptide complexes I, III, IV, and V except complex II. Twenty-two mitochondrial genes are transcribed into mitochondrial transfer RNAs (tRNAs). Two mitochondrial genes are transcribed into mitochondrial ribosomal RNAs (12S rRNA and 16S rRNA). These tRNAs and rRNAs together ensure the normal operation of mitochondrial gene expression (Fig. 1). The yeast strain *Saccharomyces cerevisiae* has about 1000 mitochondrial proteins.^14^^,^^15^ Yeast mtDNA can encode eight proteins including cytochrome c oxidase subunit I/II/III, ATPase 6/8/9, cytochrome b, and ribosomal protein. Yeast mitochondria lack subunits of multipolypeptide complex I. Thus, yeast OXPHOS has four multipolypeptide complexes II, III, IV and V. Seven proteins are structural subunits of multipolypeptide complexes and one is a ribosomal subunit. Yeast mtDNA encodes two rRNAs (15S rRNA and 21S rRNA) and 24 tRNAs, which are necessary for mitochondrial gene expression.

## Maintaining the integrity of mtDNA

**Figure 2:**
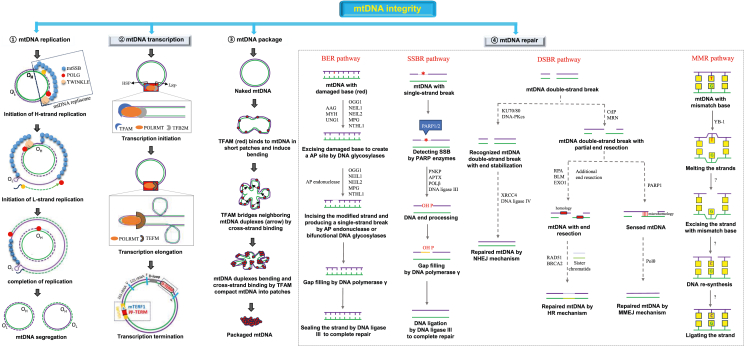
Overview of mtDNA integrity maintenance involving mtDNA replication, transcription, package, and repair. ① At the heavy-strand origin of replication, DNA polymerase-γ (POLG), mitochondrial single-stranded binding protein (mtSSB), and TWINKLE first start mtDNA replication. The replication of the light strand is triggered when the synthesis of the heavy strand is around two-thirds completed. ② mtDNA transcription is initiated by mitochondrial transcription factor B2 (TFB2M), transcription and packaging factor (TFAM), and mitochondrial DNA-directed RNA polymerase (POLRMT). The termination of mtDNA transcription occurs at the promoter-proximal transcription terminator (pp-TERM) and promoter distal transcription terminator (D-TERM). Mitochondrial transcription termination factor (mTERF) interacts with pp-TERM, thus promoting transcription termination. ③ The bending and compacting of mtDNA could be prompted by the binding of mtDNA to TFAM molecules. Finally, TFAM molecules tightly package mtDNA into mitochondrial nucleoids. ④ mtDNA might be repaired by four pathways including base excision repair (BER), single-strand break repair (SSBR), double-strand break repair (DSBR), and mismatch repair (MMR).

mtDNA integrity plays an important role in the maintenance of the respiratory chain's function as an ATP producer.^16^ Maintenance of mtDNA integrity is associated with a diversity of activities, which include mtDNA replication, transcription, package, and repair, aimed at preserving the normal function of mtDNA molecules (Fig. 2).

### mtDNA replication

The number of mtDNA determines the functional complement of mtDNA per cell.^17^ mtDNA copy number is counted to reflect the number of mtDNA in the mitochondrial genome.^18^^,^^19^ Nuclear DNA contains only two copies per cell. In contrast, there can be hundreds or even thousands of mtDNA copies in a single cell, which depends on tissue origins and different types of tissue cells.^18^^,^^20^^,^^21^ In humans, the mtDNA copy number of myocardial tissues is approximately 6970 copies per cell, which is almost twice that of skeletal muscle cells (about 3650).^17^ In mice, the heart, skeletal muscle, and liver contain respectively 3956, 3199, and 2987 copies per cell, while the spleen contains significantly less, namely 251 copies per cell.^22^ These data suggest that high-energy-consuming tissues acquire more copies of mtDNA in order to produce larger amounts of ATP. Multiple evidence shows that many diseases such as aging and cardiovascular diseases are related to mtDNA copy number loss.^23^ Therefore, keeping the normal levels of mtDNA copy number in tissue cells is necessary to maintain cell functions.

The replication of mtDNA is a critical mechanism for reconstituting the mtDNA pool. mtDNA replication is different from nuclear DNA replication. mtDNA replication is beyond the limitation of the cell cycle in replisomes (DNA/protein complex).^16^ DNA polymerase-γ (POLG), mitochondrial single-stranded binding protein (mtSSB), and the replicative hexameric helicase TWINKLE are the core subunits of mtDNA replisome.^15^^,^^24^ They first initiate mtDNA replication at the heavy-strand origin of replication, followed by the full-length nascent heavy-strand formation.^25^^,^^26^ The replication of the light strand is activated after about two-thirds of the heavy strand has been synthesized.^25^^,^^26^ The only known DNA polymerase within mitochondria up to now is POLG.^22^^,^^27^ POLG holoenzyme is a heterodimeric complex that consists of one catalytic subunit (POLGA) responsible for checking through the newly synthesized DNA strand, and two accessory subunits (POLGB) for maintaining enzymatic stability and efﬁciency.^22^^,^^28^^,^^29^ TWINKLE is regarded as the core helicase in mitochondria. Besides, no other helicase is needed for mtDNA replication. Its function in mtDNA replication is to unwind the double-stranded DNA.^25^^,^^26^ mtSSBP can bind to the single-stranded DNA. The secondary structures of the nascent DNA strand were decreased by mtSSBP to benefit replication.^30^ Beyond its interaction function with DNA, mtSSBP can help POLG to increase the processivity of DNA strand elongation and activate TWINKLE at the location of the mitochondrial replication fork.^25^^,^^26^^,^^31^ Besides the above core proteins, other proteins associated with mtDNA replication are DNA ligase III, the mitochondrial genome and maintenance exonuclease 1,^32^ and RNAse H1.^24^^,^^32^ In addition, since transcription and replication are closely related in mitochondria, transcription-related proteins such as mitochondrial DNA-directed RNA polymerase (POLRMT),^33^ transcription elongation factor (TEFM),^30^ transcription and packaging factor (TFAM),^14^ mitochondrial transcription factor B2 (TFB2M),^33^ mitochondrial transcription termination factors (mTERF1 and mTERF3)^34^, ^35^, ^36^, ^37^ are also involved in mtDNA replication.^23^^,^^38^ The functions of these proteins are listed in Table 2.

**Table 2:** Main molecules related to the replication, package and transcription of mtDNA.

Molecules	mtDNA replication	mtDNA package	mtDNA transcription	References
POLG POLGA POLGB	Proofreading the newly synthesized DNA strand Maintaining enzymatic stability and efﬁciency	The component of mito. nucleoid	/	22,28,29
TWINKLE	Unwinding the double-stranded DNA	The component of mito. nucleoid	/	25,26
mtSSB	• Binding to the single-stranded DNA, decreasing secondary structures of the nascent DNA strand for replication • Increasing the processivity of POLG in DNA strand elongation • Stimulating the activity of TWINKLE at the mitochondrial replication fork	The component of mito. nucleoid	/	25,26,30,31
MGME1	Cleaving ﬂaps for the removal of the RNA primers at the origins of DNA replication, to enable efﬁcient ligation of newly replicated DNA strands	/	/	32
DNA ligase III	Ligating the newly formed DNA strands	/	/	24,32
RNAse H1	Removing RNA primers at the origin of the heavy strand and the origin of the light strand	/	/	24,32
POLRMT	Synthesizing primers at both heavy strand and light strand of DNA	The component of mito. nucleoid	Having DNA-dependent RNA polymerase activity and binding speciﬁcally to promoter elements of mtDNA	11,14,33,39
TFAM	A limiting determinant of mtDNA replication	Binding directly to mtDNA to coat mtDNA	Binding to mtDNA's promoters and recruits POLRMT to the transcription start site	14,15,52
TEFM	• Preventing the generation of mtDNA replication primers • Forming a sliding clamp to increase the processivity of POLRMT	The component of mito. nucleoid	Binding to the nontemplate strand of the transcription bubble for transcription elongation	11,30,39
TFB2M	the priming of not only strand-asynchronous but also strand-coupled replication	The component of mito. nucleoid	Facilitating melting of the promoter to permit the initiation of RNA synthesis	23,33,38
mTERF1		The component of mito. nucleoid	Specifying site-speciﬁc transcription termination	34, 35, 36, 37
mTERF3		The component of mito. nucleoid	Acting as a promoter-proximal transcription termination factor to negatively regulate mtDNA transcription initiation	34, 35, 36, 37

### mtDNA transcription

The transcriptional apparatus in mitochondria is a complex system associated with multiple components. The main components of this system in mammalian mitochondria comprise POLRMT and several accessory transcription factors TFAM, TFB2M, and TEFM.^11^^,^^39^ All of these main members are imported into mitochondria since they are encoded by nuclear genes. These components work together to initiate transcription from mtDNA promoters. POLRMT has DNA-dependent RNA polymerase activity and is at the heart of the mtDNA transcription system. POLRMT can bind to promoter elements speciﬁcally; however, it can only exert its transcription function with the assistance of TFAM, TFB2, and TEFM.^15^^,^^40^ TFAM is a known mtDNA transcription factor.^41^^,^^42^ It binds to the light and heavy strand promoters of mtDNA with high affinity separately. After the promoters interact with TFAM, POLRMT and TFB2M are recruited to the transcription start site. TFB2M permits the initiation of RNA synthesis by facilitating the melting of the promoter. In other words, the normal transcriptional effect of TFAM requires the presence of TFB2M.^11^^,^^41^ TEFM improves the stability of POLRMT combining with the template by associating with POLRMT.^43^

The steady-state levels of mtDNA transcripts are tightly regulated by certain termination events. There are two distinct transcription terminations of mtDNA on the genome in mammalian cells.^44^ Beyond the 16S rRNA gene is the first site of termination that is localized to the tRNAL (UUR) gene. This terminator is called promoter-proximal transcription terminator (pp-TERM). Pp-TERM binds to mitochondrial transcription termination factor (mTERF). The mTERF promotes transcription termination.^14^^,^^34^^,^^43^ The family of mammalian mTERF has four members, named MTERF1 to MTERF4. It has been shown that mTERF1 and mTERF3 mediate mtDNA transcription. mTERF1 promotes transcription termination by binding to the promoter-proximal mt-TERM sequence.^44^ mTERF3 associates with the promoter region of mtDNA and acts as a promoter-proximal transcription termination factor to negatively regulate mtDNA transcription initiation.^34^^,^^45^ Beyond the three-stranded DNA loop area, just upstream of the tRNAPhe gene, is the second point of termination, known as the promoter distal transcription terminator (D-TERM).^44^ In contrast, the mechanism of transcription termination for mtDNA L-strand remains less well studied. Based on the fact that the sequence of the L-strand transcription terminator exhibits bi-directional activity, it seems that mt-TERM may also be involved in this termination site.^44^^,^^46^ Also, it is inferred that D-TERM exists in the mtDNA L-strand.^43^^,^^47^ Further investigation is required to determine whether mt-TERM and D-TERM are involved in transcription termination of mtDNA L-strand, and the underlying molecular mechanisms.

### mtDNA package

mtDNA has been proven not to be naked. It is packaged into a nucleoid structure with a group of protein factors for providing stability and protection against the attacks of harmful stimuli.^23^^,^^48^ The average diameter of mitochondrial nucleoids is about 100 nm. The majority of nucleoids in mammalian mitochondria typically only have one copy of mtDNA.^43^^,^^49^ However, in human cardiac tissue, mtDNAs that are multimeric and contain more complex junctions are found in nucleoids.^43^^,^^50^^,^^51^

The mitochondrial replication machinery is composed of POLG, TWINKLE, and mtSSB. In addition, POLRMT, TFAM, TFB2M, and TEFM together form the mitochondrial transcription machinery. It is found that mitochondrial nucleoid contains the key components of the replication machinery and the transcription machinery. What's more, chaperones, proteases, RNA-binding proteins, mitochondrial ribosomal proteins, and RNA processing proteins are also identified in nucleoids.^52^ Although mitochondrial nucleoid has numerous proteins, the only protein responsible for the mtDNA package is TFAM.^15^^,^^52^ TFAM consists of two high-mobility group-box domains and belongs to the high-mobility group proteins. TFAM can associate with throughout mtDNA in a nonspecific DNA-binding manner. The binding ratio of TFAM/mtDNA is about 900:1. It means that one molecule of mtDNA is bound to about 900 molecules of TFAM on average.^41^ TFAM is abundantly expressed in mitochondria. Therefore, the mtDNA could be enveloped by this amount of TFAM entirely.

### mtDNA repair

mtDNA is multi-copied, unlike nuclear DNA, and has a comprehensive scavenging system for free radicals. Therefore, mtDNA was once believed short of DNA repair mechanisms, even though it is frequently noted that mitochondria are key sites for free radical presence.^53^^,^^54^ However, numerous mtDNA repair mechanisms have been proposed according to decades of research on mitochondria, such as single-strand break repair (SSBR), base excision repair (BER), mismatch repair (MMR), and double-strand break repair (DSBR).^24^^,^^53^

Due to the close proximity of mtDNA to reactive oxygen species generated by the electron transport chain, oxidative damage is thought to be one of the main types of mtDNA damage. Thus, BER is mainly responsible for repairing mtDNA damage. BER consists of three main processes. (i) The first is the identification of the damaged DNA base and executing excision, which is initiated with monofunctional and bifunctional DNA glycosylases. To date, three monofunctional DNA glycosylases have been characterized, including MutY glycosylase homologue, alkyladenine DNA glycosylase, and uracil N-glycosyalse 1, and five bifunctional DNA glycosylases have also been identified, including Nei-like 1, 8-oxoguanine DNA glycosylase-1 (OGG1), N-methylpurine DNA glycosylase, Nei-like 2, and Nth-Like 1.^54^^,^^55^ The other two main processes are (ii) apurinic/apyrimidinic site removal by bifunctional DNA glycosylases or apurinic/apyrimidinic endonuclease III and (ii) gap filling and ligation by DNA polymerase γ and DNA ligase III respectively.^54^^,^^56^

In the range of 10^4^∼10^5^ times per cell each day, SSBR emerges widely in cells.^57^ It means that every 1–10 s there will be one SSB in each cell. If these SSBs are not rapidly repaired, it inevitably results in SSB accumulation, thereby inducing the dysfunction of mtDNA transcription and replication. SSBR has three basic steps^53^^,^^57^: (i) single-strand break detection by PARP enzymes including PARP1 and PARP2; (ii) DNA end processing by DNA ligase III, APTX, POLβ, and PNKP; (iii) gap filling and DNA ligation. The same enzymes utilized for BER are used in SSBR for gap filling and DNA ligation.

In mammalian nuclei, the most hazardous type of DNA damage is considered to be DSBs, as they can cause the loss of substantial chromosomal regions. Three pathways are related to nuclear DSB repair: microhomology-mediated end joining, homologous recombination, and nonhomologous end joining.^24^^,^^53^^,^^58^^,^^59^ The nonhomologous end-joining pathway is mediated by XRCC4, DNA-PKcs, Ku70/80, and DNA ligase IV; the homologous recombination pathway requires the recombinase RAD51 and error-prone polymerases (RPA and BRCA2) to repair DSBs; and microhomology-mediated end joining is controlled by PARP-1 and Polθ. Notably, so far there is no strong proof for the existence of efficient DSBR in mammalian mtDNA. However, some mediated DSBR factors have been identified within mitochondria such as Ku80,^60^ XRCC4,^61^ and RAD51.^62^ It seems that mammalian mtDNA might be capable of DSBR. Of course, further investigations need to be done in order to get conclusive evidence.

MMR aims at the removal of mismatched nucleotides. MutS protein homology 2/3/6 (MSH2/3/6), MLH1, PMS1 homology 2, and YB-1 are key proteins composing the nucleus MMR system.^63^^,^^64^ However, only YB-1 is found to participate in mtDNA repair.^64^^,^^65^ To generate MutSα and MutSβ complexes, MSH2 associates with MSH6 or MSH3 separately.^64^ MSH2, MSH3, and MSH6 have not been identified in mitochondria, despite MutSα and MutSβ could be found in MMR activity of mitochondria.^64^

## The effect of mtDNA interference on cardiomyocytes at the basal level

It is established that the deficiency of mtDNA or the disruption of mtDNA integrity induces mitochondrial dysfunction and cardiomyocyte injury at baseline. mtDNA depletion by ethidium bromide reduces ATP production and increases cardiomyocyte death.^66^ mtDNA deletions causing Kearns-Sayre syndrome can result in a dilated cardiomyopathy characterized by the deficient respiratory chain in the form of a mosaic pattern.^67^^,^^68^ Mice carrying a proofreading defective POLG are found to have a significant increase in heart weight along with the decrease of mtDNA-encoded complex I and IV subunits.^69^ Conditional knockout POLRMT mice display developing left ventricular chamber dilation and heart enlargement. Furthermore, the heart rate variability decreases. Complexes I, IV, and V but not the complex II encoded by the nucleus show impaired enzyme activities in POLRMT-deficient hearts.^70^ Heart size and weight are enhanced in cardiac-specific TFAM knockout (Tfam^loxP^/Tfam^loxP^, +/Ckmm-cre) mice, along with left ventricular chamber dilation; correspondingly, the expression of mtDNA-encoded ATP8 protein is down-regulated and the mtDNA copy number is reduced in Tfam^loxP^/Tfam^loxP^, +/Ckmm-cre hearts. Concomitantly, mitochondrial respiratory chain function in Tfam^loxP^/Tfam^loxP^, +/Ckmm-cre heart is weakened. This is reflected by the decline of complexes I and IV activities. These complexes consist of mtDNA-encoded subunits. In contrast, the activity of complex II is normal.^68^^,^^71^ In mouse hearts of cardiac-specific OGG1 overexpression, the levels of mitochondrial 8-OHdG are lowered under basal conditions,^72^ whereas deficiencies in OGG1 elevate the levels of mitochondrial 8-OHdG in myocardial tissues.^73^ OGG1 overexpression decreases transaortic constriction-induced cardiac fibrosis and improves myocardial function.^72^ These data indicate that mtDNA integrity is required for mitochondrial function and cardiomyocyte survival at baseline.

## mtDNA integrity is disrupted in cardiomyocytes upon I/R

**Figure 3:**
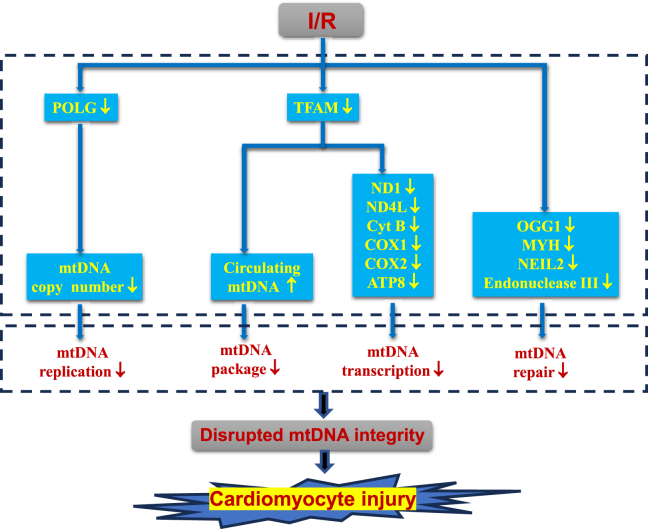
Summary of the molecular mechanisms by which I/R disrupts mtDNA integrity, thereby inducing cardiomyocyte injury. I/R reduces the copy number of cardiomyocyte mtDNA by the down-regulation of DNA polymerase-γ (POLG) expression, which results in the decrease of mtDNA replication. The down-regulation of TFAM expression induced by I/R increases the level of circulating mtDNA, indicating that the mtDNA package is impaired. Also, TFAM down-regulation in cardiomyocytes upon I/R is accompanied by a decrease in mtDNA-encoded genes and main molecules mediating mtDNA repair. It means that the ability of mtDNA transcription and repair is reduced under I/R conditions. These changes in mtDNA replication, transcription, package, and repair lead to the disruption of mtDNA integrity, inevitably inducing cardiomyocyte injury.

Available evidence indicates that myocardial I/R leads to mtDNA integrity disruption.^74^, ^75^, ^76^ mtDNA integrity disruption includes the decrease of mtDNA transcription level and copy number, as well as the increase of the mtDNA damage which results in mitochondrial dysfunction, thereby inevitably leading to myocardial injury (Fig. 3 and Table 3).

**Table 3:** The disruption of mtDNA integrity in cardiomyocytes under I/R conditions.

Disruption of mtDNA integrity	Changes of mtDNA	Molecules involved in mtDNA damage	Changes of mitochondrial functions	Myocardial injury	References
mtDNA replication ↓	mtDNA copy number ↓ mtDNA content ↓	TFAM ↓ POLG ↓	ATP level ↓ Activity of complexes I–V ↓ VDAC ↓ ΔΨm ↓ Mito. ROS ↑	Cell apoptosis ↑ Infarction area↑ CK-MB and cTnI ↑ Heart functions ↓	77, 78, 79, 80, 81, 82, 83, 84, 85, 86, 87
mtDNA transcript ↓	Expression levels of mtDNA-coding genes including ND1, ND3, ND4, ND4L, CytB, COX1, COX2, ATP6, ATP 8 and Cyt B ↓	TFAM ↓ TFB2M ↓	ATP level ↓ Activity of complexes I–V ↓ VDAC ↓ ΔΨm ↓ Mito. ROS ↑	Cell apoptosis ↑ Infarction area↑ CK-MB and cTnI ↑ Heart functions ↓	77,84,86, 87, 88, 89, 90, 91
mtDNA package and mtDNA repair ↓	Segment mtDNA ↑ Plasma mtDNA levels ↑	TFAM ↓ OGG1↓ MYH ↓ NEIL2 ↓ Endonuclease III ↓ 8-OHdG ↑	ATP level ↓ Activity of complexes I–V ↓ VDAC ↓ ΔΨm ↓ Mito. 8-OHdG ↑ Mito. ROS ↑	Inflammatory response ↑ Cell apoptosis ↑ Infarction area↑ CK-MB and cTnI ↑ Heart functions ↓	77,80,84,86,87,89, 90, 91,93,94

TFAM: transcription and packaging factor; POLG: DNA polymerase-γ; VDAC: voltage-dependent anion channel; ΔΨm: mitochondrial membrane potential; ROS: reactive oxygen species; CK-MB: creatine kinase-MB; cTnI: cardiac troponin; ND: NADH dehydrogenases; COX: cytochrome c oxidase; Cyt B: cytochrome B; TFB2M: mitochondrial transcription factor B2; OGG1: 8-oxoguanine DNA glycosylase-1; MYH: glycosylase homologue; NEIL2: Nei-like 2.

### I/R reduces mtDNA transcription level and copy number in cardiomyocytes

mtDNA copy number is one of the major factors that influence mitochondrial function. It has been confirmed that I/R reduces mtDNA copy number. In mouse/rat hearts *in vivo*, it is found that I/R induces mtDNA copy number decreasing in the myocardium, along with impaired mitochondrial and heart functions.^77^, ^78^, ^79^, ^80^, ^81^, ^82^, ^83^ I/R hearts *ex vivo* induced by the Langendorff perfusion system show a significant decline in the mtDNA copy number.^84^^,^^85^ Also, the mtDNA copy number decreases accompanied by increased cardiomyocyte death in cultured cardiomyocytes exposed to hypoxia/reoxygenation.^78^, ^79^, ^80^^,^^83^ POLG is the core subunit of the mtDNA replisome. According to reports, myocardial I/R suppressed the POLG protein's expression.^77^^,^^84^^,^^86^^,^^87^

In the electron transport chain, 22 tRNAs and 2 rRNAs along with 13 structural subunits of multipolypeptide complexes I, III, IV, and V are all encoded by mtDNA. Hence, any mtDNA damage may lead to mitochondrial transcription dysfunction or even oxidative phosphorylation dysfunction. The expression of 13 electron transport chain genes encoded by mtDNA is analyzed, and ATP8, cytochrome c oxidase subunit I, cytochrome c oxidase subunit II, Cyt B, ND4L, and ND1 are down-regulated by I/R in rat hearts. As a result of the down-regulation of these genes, electron transport chain complexes I, III, IV, and V are less active, which consequently declines ATP levels and raises reactive oxygen species.^84^^,^^86^^,^^88^ TFAM is one of the major transcription factors which mediate mtDNA transcription. The expression level of TFAM is down-regulated in I/R exposed cardiomyocytes, accompanied by a decrease in mtDNA-encoded genes.^77^^,^^84^^,^^86^^,^^87^^,^^89^, ^90^, ^91^ The above findings suggest that the transcriptional levels of mtDNA are down-regulated and TFAM might mediate the change. However, how does TFAM mediate the I/R-induced decrease of mtDNA transcriptional levels? Are there any other transcription factors such as POLRMT, TFB2M, and TEFM involved in the changes of mtDNA transcriptional levels under I/R conditions? These issues still need to be determined.

### The ability of mtDNA package and repair is impaired in cardiomyocytes exposed to I/R

TFAM is an mtDNA packing protein in addition to an mtDNA transcription factor. One molecule of mtDNA is wrapped by approximately 900 molecules of TFAM. Under I/R conditions, TFAM expression is down-regulated in cardiomyocytes.^77^^,^^84^^,^^86^^,^^87^^,^^89^, ^90^, ^91^ It means the package of mtDNA is impaired due to not having enough TFAM to coat mtDNA. Furthermore, mtDNA leaks into the cytosol, if encouraged by abnormal mtDNA packaging, where it is then released into circulation.^92^ Xie et al find that in the plasma of rats who have undergone I/R, the plasma mtDNA levels are noticeably higher.^93^ Also, according to Longnus et al, upon the emergence of ischemia, mtDNA is quickly released into the bloodstream. Meanwhile, during reperfusion, the concentration of mtDNA will increase continuously.^94^ Patients suffering from stable angina pectoris or with ST-segment elevation acute myocardial infarction display increased circulating mtDNA when they are treated with percutaneous coronary intervention.^95^^,^^96^

mtDNA damage can be indicated by measuring the proportion of long versus short PCR products. To investigate whether mtDNA damage occurs in cardiomyocytes upon I/R, Andres et al compared short versus long mtDNA amplification products in myocardial tissues from patients undergoing coronary arterial bypass grafts with PCR analysis.^97^ The long PCR product consistently decreased, indicating that I/R induces bulky adducts or single-/double-strand breaks in mtDNA. Similarly, in isolated hearts from I/R-exposed mice, mtDNA damage occurs.^74^^,^^80^^,^^98^^,^^99^ A biomarker of DNA oxidative damage is 8-OHdG, a deoxyguanosine derivative that has undergone oxidation. In mitochondria of I/R-treated rat cardiomyocytes, the presence of 8-OHdG-positive cells notably increases, indicative of the occurrence of mtDNA oxidative damage.^66^ If damaged mtDNA is not repaired, they accumulate to block transcription and replication of mtDNA.^5^ However, emerging studies show that the ability of mtDNA repair is reduced in cardiomyocytes exposed to I/R. The expression changes of DNA glycosylases involved in BER are detected by Bliksøen et al in isolated hearts subjected to I/R, and it is found that OGG1, MutY glycosylase homologue, and Nei-like 2 are down-regulated, accompanied by the increase of mtDNA damage.^74^ Knockout of OGG1 in mice further aggravates mtDNA damage. The protein level of the DNA glycosylase/apurinic/apyrimidinic lyase, endonuclease III, is decreased in a rat's myocardial I/R exposure model, suggesting that mtDNA repair is weakened.^5^ An intravenous bolus of endonuclease III just before reperfusion prevents the release of damaged mtDNA from cardiomyocytes and reduces infarct size. The above findings confirm that the collapse of the BER mechanism is involved in I/R-induced mtDNA damage in cardiomyocytes. mtDNA repair mechanisms are reported to include BER, SSBR, DSBR, and MMR. However, whether SSBR, DSBR, and MMR mechanisms also collapse in cardiomyocytes upon I/R insults remains unknown.

### The damaged mtDNA released from mitochondria aggravates myocardial I/R injury and causes inflammatory responses

The damaged mtDNA not only accumulates in mitochondria to interfere with the transcription and replication of mtDNA but is also released into the circulation to aggravate tissue injury.^100^ The reperfused myocardium may suffer a wave of necrosis due to released mtDNA fragments. However, eliminating the released fragments of mtDNA with a DNase I intravenous injection significantly abrogates myocardial infarct size upon I/R.^5^^,^^101^^,^^102^ Myocardial infarct size and cardiomyocyte apoptosis upon I/R are both significantly increased after the injection of mtDNA fragments.^93^^,^^102^ mtDNA contains unmethylated CpG motifs. The oxidatively damaged adducts and unmethylated CpG motifs containing damaged mtDNA leak from mitochondria and contribute to the pro-inflammatory effects.^103^^,^^104^ The damaged mtDNA is encouraged to be released by I/R. The released mtDNA induced by I/R activates signaling cascades resulting in nuclear transcription of pro-inﬂammatory cytokines that aggravate I/R injury.^7^^,^^102^^,^^105^ Cardiopulmonary bypass triggers mtDNA release that activates Toll-like receptor 9 and induces the release of IL-6 in turn.^106^ mtDNA up-regulates pro-inflammatory cytokine levels, such as TNFα and IL-1β, in cardiomyocytes that have undergone hypoxia-reoxygenation.^107^ The inflammatory response induced by the released mtDNA causes inflammatory myocardial injury.

## mtDNA, a new target for I/R-induced myocardial injury therapy

Inhibiting the damage of mtDNA might be a novel way to treat myocardial injury caused by I/R since conclusive data demonstrates that aberrant mtDNA is involved in I/R-induced myocardial injury (Table 4). The heart has a high expression of Perm1 (PGC-1 and ERR-induced regulator, muscle 1). Perm1 is a mediator of mitochondrial biogenesis.^108^ Overexpression of Perm1 up-regulates the expression levels of cytochrome c oxidase subunits II and III encoded by mtDNA, increases the copy number of mtDNA, and reduces cardiomyocyte death caused by I/R.^83^ An adipokine named C1q/tumor necrosis factor-related protein-3 is able to promote mtDNA-coded gene expression and ATP content to shield cardiomyocytes from I/R-induced damage.^90^ Beclin1 and suberoylanilide hydroxamic acid, a histone deacetylase inhibitor approved by the FDA, are powerful autophagy-inducing proteins. Both have been shown to increase mtDNA content and reduce mtDNA damage, thereby lowering the size of myocardial infarction when given just following reperfusion.^80^^,^^81^ Mitochondrial complex I is central to the pathological reactive oxygen species production in I/R-treated hearts.^109^ A single point mutation in a mtDNA-encoded subunit of complex I still functions normally for NADH oxidation, but is unable to catalyze reactive oxygen species production by reverse electron transport. Therefore, the complex I mutation is protective against I/R-induced myocardial injury.^109^ I/R can stimulate mtDNA methylation, and mtDNA hypermethylation has a negative correlation with mtDNA coding and transcript functions.^84^^,^^86^^,^^88^ Targeting mtDNA methylation via its inhibitor, 5-azacytidine, improves mitochondrial function and inhibits I/R-initiated cardiomyocyte apoptosis.^86^^,^^87^ Endo III glycosylase/apurinic/apyrimidinic lyase containing fusion protein exscien1-III plays an essential role in repairing mtDNA damage. The myocardial infarct size is reduced when exscien1-III is injected intracardiacally into the left ventricular lumen at the time of reperfusion.^99^ Administration of anti-oxidant MitoQ, lycopene, or the senolytics dasatinib plus quercetin reduces mtDNA damage and the release of damaged mtDNA, thereby inhibiting inflammatory response and cardiomyocyte injury upon I/R.^7^^,^^66^^,^^98^ Nucleolin can bind to mtDNA to mediate mtDNA pro-inflammatory effects. Treatment of cardiomyocytes with midkine blocking the binding of mtDNA to nucleolin or AS1411, the nucleolin inhibitor, suppresses I/R-induced inflammatory response.^107^ These data suggest that the maintenance of mtDNA integrity can effectively relieve myocardial injury triggered by I/R.

**Table 4:** Treatment targeting mtDNA for mitochondrial dysfunctions and myocardial injury induced by I/R.

Therapeutic strategies	Types of models	Intervention methods	Changes of mtDNA integrity	Indexes changes of mtDNA integrity	Mechanisms	Changes of mitochondrial functions	Myocardial injury	Refe.
Perm1	*in vitro*	Treating cells with adPerm1	mtDNA replication ↑ mtDNA transcript ↑	mtDNA copy number ↑ COX 2 and COX3 ↑	TFB2M ↑	Mito. biogenesis and function ↑	Cell apoptosis ↓	83
CTRP3	*in vitro*	Treating cells with recombinant globular CTRP3	mtDNA replication ↑ mtDNA transcript ↑	mtDNA copy number ↑ Cyt B ↑	TFAM ↑	Mito. Biogenesis ↑ Activity of complexes III and V ↑ ATP level ↑	–	90
Beclin 1	*in vivo* *in vitro*	Intravenous Beclin 1/treating cells with Beclin 1	mtDNA replication ↑ mtDNA damage ↓	mtDNA content ↑ mtDNA copy number ↑	Autophagy ↑	ΔΨm ↑ ATP level ↑ Mito. Biogenesis ↑	Cell death ↓ Infarction area ↓	80
SAHA	*in vivo* *in vitro*	Intraperitoneally injecting SAHA/treating cells with SAHA	mtDNA replication ↑ mtDNA transcript ↑ mtDNA damage ↓	mtDNA content ↑ mtDNA copy number ↑ COX 2 and ATP6 ↑ Segment mtDNA ↓	Autophagy ↑	Mito. Biogenesis ↑ Activity of complexes IV ↑ VDAC ↑ ΔΨm ↑ Mito. ROS ↓	Cell apoptosis ↓ Infarction area ↓	80,81
5-azacytidine	*in vivo* *in vitro*	Intraperitoneally injecting 5-azacytidine	mtDNA replication ↑ mtDNA transcript ↑	mtDNA copy number ↑ ND1, ND3 and ND4 ↑ COX1and COX2 ↑ Cyt B and ATP6 ↑	TFAM ↑ DNA methylation ↓	Activity of complexes III and V ↑ ATP level ↑ Mito. ROS ↓	Cell apoptosis ↓ Infarction area ↓ CK-MB ↓ Heart function ↑	86,87
Exscien1-III	*in vivo* *in vitro*	Injection Exscien1-III into the LV lumen	mtDNA damage ↓	Segment mtDNA ↓	OGG1 ↑	Mito. antioxidant ↑	Cell apoptosis ↓ Infarction area ↓ Heart function ↑	99
Dasatinib plus Quercetin	*in vivo*	Peros	mtDNA damage ↓	Damaged mtDNA release ↓	–	–	Cell survival ↑ Inflammatory response ↓	7
MitoQ	*in vivo*	Flushing hearts with MitoQ	mtDNA damage ↓	Segment mtDNA ↓	–	Mito. ROS ↓	cTnI ↓ Inflammatory response ↓	98
Lycopene	*in vivo* *in vitro*	Peros or incubation of cultured cardiomyocytes with lycopene	mtDNA replication ↑ mtDNA transcript ↑ mtDNA damage ↓	mtDNA copy number ↑ mtDNA content ↑ ND1 and COX I ↑ Mito. 8-OHdG ↓	TFAM ↑	ATP level ↑ ΔΨm ↑ Mito. ROS ↓	Cell apoptosis ↓ Infarction area ↓ CK-MB and cTnI ↓	66
Midkine/AS1411	*in vitro*	Treating cells with Midkine/AS1411	mtDNA damage ↓	Segment mtDNA ↓	Blocking the binding of mtDNA to nucleolin	–	Inflammatory response ↓	107
Atractylenolide I	*in vivo* *in vitro*	Intraperitoneal injection	mtDNA replication ↑	mtDNA copy number ↑	–	ΔΨm ↑ Mito. ROS ↓	Cell apoptosis ↓ Infarction area ↓ CK-MB and cTnI ↓	79
Sappanone A	*in vivo*	Intracoronary infusion using the Langendorff	mtDNA replication ↑	mtDNA copy number ↑	–	Mito. Biogenesis ↑ ATP level ↑ ΔΨm ↑ mPTP opening ↓ Mito. ROS ↓ Activity of complexes I–IV ↑	Cell death ↓ Infarction area ↓ CK-MB and cTnI ↓	85
Salvianolate	*in vivo*	intravenous injection	mtDNA damage ↓	Mito. 8-OHdG ↓	–	ATP level ↑	Cell apoptosis ↓ Infarction area ↓ CK and cTnI ↓	112
Fisetin	*in vivo*	Intraperitoneal injection	mtDNA replication ↑ mtDNA transcript ↑ mtDNA damage ↓	mtDNA copy number ↑ ND1, ND2, ND4, CytB, COX1, COX2 and ATP6 ↑	POLG ↑ TFAM ↑	ATP level ↑	Cardiac function↑	113
Huoxue Huatan Decoction	*in vivo*	Peros	mtDNA replication ↑	TFAM ↑	mtDNA copy number ↑	SOD ↑ Mito. ROS ↓	Infarction area ↓ CK-MB ↓ Heart function ↑	114

Perm1: PGC-1 and estrogen-related receptor (ERR)-induced regulator, muscle 1; COX: cytochrome c oxidase; TFB2M: mitochondrial transcription factor B2; Mito.: mitochondrial; Cyt B: cytochrome B; TFAM: transcription and packaging factor; ΔΨm: mitochondrial membrane potential; cTnI: cardiac troponin; ROS: reactive oxygen species; CK-MB: creatine kinase-MB; VDAC: voltage-dependent anion channel; SAHA: suberoylanilide hydroxamic acid; LV: left ventricular; OGG1: 8-Oxoguanine DNA glycosylase; 8-OHdG: 8-hydroxyguanine; mPTP: mitochondrial permeability transition pore; SOD: superoxide dismutase.

In addition to the above therapeutic strategies, a few researchers show interest in investigating whether Chinese material medica has a direct impact on mtDNA damage with a series of *in vitro* as well as *in vivo* experimental systems. Clinical studies have indicated the efficacy of Chinese material medica as an adjuvant therapy for myocardial infarction patients receiving percutaneous coronary intervention.^110^ *Atractylodes macrocephala* contains an active ingredient named atractylenolide I which is a sesquiterpene compound.^111^ Atractylenolide I dose-dependently increases superoxide dismutase activity and mtDNA copy number and decreases reactive oxygen species to attenuate cardiomyocyte apoptosis induced by I/R.^79^ Sappanone A, a homoisoflavanone with strong anti-inflammatory and antioxidant properties, is isolated from the heartwood of *Caesalpinia sappan* L. Pretreatment with sappanone A could increase mtDNA copy number and ATP content, along with decreasing myocardial infarct size, inhibiting cardiomyocyte death, as well as improving myocardial function.^85^ Salvianolate is a water-soluble active part of salvia miltiorrhiza, and its main component contains more than 80% magnesium acetate. It is found that salvianolate is able to inhibit cardiomyocyte apoptosis and protect mitochondrial function by minimizing mtDNA oxidative damage, thereby alleviating myocardial I/R injury.^112^ Fisetin is a bioactive favanol present in many vegetables and fruits. It is reported fisetin has the ability to improve mitochondrial function and inhibit I/R-induced myocardial injury by increasing mitochondrial copy number and the levels of mtDNA-encoded genes. Further, it is proved that fisetin up-regulates the expression of TFAM and POLG, contributing to improved mtDNA replication, transcription, and package.^113^ Since more than 20 years ago, *Huoxue Huatan Decoction* has been used in clinical settings to treat coronary heart disease of phlegm–blood stasis pattern. Its components include *Salvia miltiorrhiza* Bunge, *Ziziphus jujuba* Mill, *Allium macrostemon* Bunge, *Trichosanthes kirilowii* Maxim., *Ginkgo biloba* L., *Panax notoginseng* F.H. Chen, and *Astragalus mongholicus* Bunge. *Huoxue Huatan Decoction* can promote mtDNA synthesis by enhancing the activity of the PGC-1α–NRF1–mtTFA pathway, therefore reducing I/R-induced myocardial injury.^114^ The above studies indicate that Chinese material medica could defend cardiomyocytes against I/R injury via targeting mtDNA.

## Conclusions

Various evidence supports the idea that the integrity of mtDNA is disrupted in cardiomyocytes upon I/R. The maintenance of mtDNA integrity requires four critical links including mtDNA replication, transcription, package, and repair. It has been demonstrated that all of these links are related to the disruption of mtDNA integrity induced by I/R. It is clear that the treatment strategies by targeting mtDNA are effective for mitochondrial dysfunction and cardiomyocyte injury under I/R conditions. Our understanding of mtDNA functions in myocardial I/R injury has grown substantially thanks to the aforementioned developments, while certain important phenomena/problems still require investigation in depth. (i) Many molecules mediate the four critical links of mtDNA integrity, and there are cross-talks among these different molecules. However, less is known about how myocardial I/R affects the cross-talks. More work is required to fully analyze these intricate relationships. (ii) The myocardial I/R has been reported to cause expression variations of multiple molecules involved in the maintenance of mtDNA integrity, but more research needs to be done to determine how I/R causes these changes. Future studies are necessary to gain more comprehensive knowledge about the influences of myocardial I/R upon mitochondrial and cardiomyocyte injury as well as inspiration for the development of effective mtDNA-based remedies.

## Conflict of interests

The authors declare no conflict of interests.

## Funding

This work was supported by grants from the 10.13039/501100001809National Natural Science Foundation of China (No. 81970246, 81470437).
